# AlphaFold2 assists in providing novel mechanistic insights into the interactions among the LUBAC subunits: AlphaFold2 predictions shed light on novel understanding of the LUBAC structure

**DOI:** 10.3724/abbs.2024047

**Published:** 2024-04-23

**Authors:** Chenchen Wang, Chunying Gu, Ying Lv, Hongyu Liu, Yanan Wang, Yongmei Zuo, Guangyu Jiang, Lili Liu, Jiafu Liu

**Affiliations:** 1 College of Veterinary Medicine Northeast Agricultural University Harbin 150030 China; 2 Department of Medical Laboratory Science and Technology Harbin Medical University-Daqing Daqing 163319 China; 3 College of Life Sciences Northeast Agricultural University Harbin 150030 China; 4 Preventive and Control Center for Animal Disease of Heilongjiang Province Harbin 150069 China; 5 College of Basic Medical Sciences Harbin Medical University-Daqing Daqing 163319 China; 6 Heilongjiang Institute of Animal Health Inspection Harbin 150006 China

**Keywords:** LUBAC, LTM motif, HOIP-UBA, HOIL-1L-UBL, SHARPIN-UBL

## Abstract

The linear ubiquitin chain assembly complex (LUBAC) is the only known E3 ligase complex in which the ubiquitin-like (UBL) domains of SHARPIN and HOIL-1L interact with HOIP to determine the structural stability of LUBAC. The interactions between subunits within LUBAC have been a topic of extensive research. However, the impact of the LTM motif on the interaction between the UBL domains of SHARPIN and HOIL-1L with HOIP remains unclear. Here, we discover that the absence of the LTM motif in the AlphaFold2-predicted LUBAC structure alters the HOIP-UBA structure. We employ GeoPPI to calculate the changes in binding free energy (ΔG) caused by single-point mutations between subunits, simulating their protein-protein interactions. The results reveal that the presence of the LTM motif decreases the interaction between the UBL domains of SHARPIN and HOIL-1L with HOIP, leading to a decrease in the structural stability of LUBAC. Furthermore, using the AlphaFold2-predicted results, we find that HOIP (629‒695) and HOIP-UBA bind to both sides of HOIL-1L-UBL, respectively. The experiments of Gromacs molecular dynamics simulations, SPR and ITC demonstrate that the elongated domain formed by HOIP (629‒695) and HOIP-UBA, hereafter referred to as the HOIP (466‒695) structure, interacts with HOIL-1L-UBL to form a structurally stable complex. These findings illustrate the collaborative interaction between HOIP-UBA and HOIP (629‒695) with HOIL-1L-UBL, which influences the structural stability of LUBAC.

## Introduction

The process of covalent conjugation between proteins and several small ubiquitin (Ub) molecules is known as ubiquitination, which is a type of posttranslational modification that plays a crucial role in biological processes [1]. Ubiquitination is a three-step catalytic reaction process sequentially catalyzed by a Ub-activating enzyme (E1), a Ub-conjugating enzyme (E2), and a Ub ligase (E3) [ 2, 3] . The linear ubiquitin chain assembly complex (LUBAC) is the only known E3 ligase complex which is composed of the catalytic subunit HOIP and two accessory subunits, HOIL-1L and SHARPIN [4]. In LUBAC, the catalytic subunit HOIP interacts with the ubiquitin-like (UBL) domains of SHARPIN and HOIL-1L to maintain the stability of the structure and function of LUBAC [5]. Dysfunction or impairment of any of the three subunits of LUBAC can lead to inflammation, immune deficiency, and even death in animals and humans [6].

HOIP is an E3 ligase of the RBR (RING-between-RING) type [7]. Its full length consists of 1072 residues, and its structure mainly includes an N-terminal PNGase/UBA or UBX-containing protein (PUB) domain, followed by a B-box type zinc finger (ZF), a typical ZF, two Nlp4-like ZF domains (NZF1 and NZF2), an intermediate atypical Ub-associated domain (UBA), and a C-terminal RBR domain conjugated with a unique linear Ub chain determining domain (LDD) [8]. The RBR and LDD regions together form the catalytic core of HOIP, which is responsible for linear Ub chain assembly [9]. Free HOIP exists in a partially autoinhibited state, where the UBA domain of HOIP cannot recognize Ub chains, and the biological activity of the RBR-LDD domain is inhibited [10]. SHARPIN and HOIL-1L are accessory subunits that form LUBAC with HOIP. Binding of HOIL-1L or SHARPIN to HOIP releases the autoinhibition of HOIP [11].

HOIL-1L and SHARPIN, as accessory subunits, participate in the formation of LUBAC along with the catalytic subunit HOIP [12]. HOIL-1L is composed of an N-terminal LTM motif, a UBL domain, an intermediate NZF domain, and a C-terminal RBR domain [13]. Like HOIL-1L, SHARPIN also contains highly similar amino acid sequences and includes LTM, UBL, and Npl4 zinc finger (NZF) domains [14]. However, unlike HOIL-1L, SHARPIN possesses a unique N-terminal PH domain (residues 1‒127) in addition to the aforementioned domains [15].

The auxiliary subunit HOIL-1L interacts with the main catalytic subunit HOIP through its ubiquitin-like (UBL) domain, which binds to the UBA domain of HOIP. However, the ternary crystal structure of LUBAC suggested that HOIL-1L and SHARPIN also interact with each other in a unique way. The LTM-mediated changes in the heterodimeric regions of HOIL-1L-UBL and SHARPIN-UBL contribute to the stability of the LUBAC core structure [ 16‒ 18] . To investigate the impacts of the LTM motif and other structures on the stability of the LUBAC structure, AlphaFold-Multimer was used to predict the structure of the LUBAC core domain without the LTM motif. Then, GeoPPI software was used to calculate the effect of amino acid mutations on protein-protein interactions in the predicted structure. Quantitative analyses were conducted to compare the interactions between different structures and their effects on the stability of the LUBAC complex.

AlphaFold, developed by DeepMind, a subsidiary of Google, is an artificial intelligence (AI) system [ 19‒ 23] . The upgraded version, AlphaFold2, is a deep learning system that combines evolutionary, physical, and geometric constraints based on protein structure in its training program [ 24, 25] . By inputting the amino acid sequence of an unknown protein structure, AlphaFold2 computes the 3D coordinates and structural files for all atoms of the input protein directly [26]. The latest version, AlphaFold 2.3.2, exhibits unprecedented accuracy in modelling protein complexes [27]. Furthermore, AlphaFold2 has been increasingly employed for the discovery of novel protein-protein interactions [28].

The ΔG is commonly used to measure the thermodynamics of protein-protein interactions [29]. However, existing experimental methods cannot comprehensively explore the impact of a large number of mutations on binding affinity within a short time frame. Therefore, utilizing fast and cost-effective computer simulations to assess the changes in binding affinity (ΔG) after mutation is a powerful alternative in protein engineering. In this study, a novel framework called GeoPPI was employed to simulate the effects of mutations on binding affinity by learning geometric representations from protein structures. Compared to traditional methods, GeoPPI has the following advantages. (1) GeoPPI can automatically learn meaningful features from protein structures for prediction without the need for complex feature engineering. (2) GeoPPI exhibits better generalizability because the geometric encoder captures the shared geometric features among different protein complexes. (3) Compared to existing methods that rely on computationally intensive biophysical simulations, GeoPPI is more efficient and accurate during the prediction phase [18].

In the present study, GeoPPI was employed to identify alterations in binding affinity resulted from single-point mutations to alanine based on the predicted structures of the LUBAC complex with varying lengths generated by AlphaFold2. The effects of single-point mutations on binding affinity were assessed across various structural states. The SPR results further validated the decreased binding affinity between HOIP-UBA in the presence of the LTM domain and either HOIL-1L-UBL or SHARPIN-UBL within the LUBAC complex. A robust interaction between HOIP (629‒695) and HOIL-1L-UBL was discovered by the prediction of the structure of the AlphaFold2 complex. This discovery was subsequently corroborated through molecular dynamics simulations conducted with Gromacs [ 30, 31] . Furthermore, SPR molecular kinetic and ITC experiments confirmed that HOIP (629‒695) enhances the interaction between HOIP and HOIL-1L-UBL, playing a crucial role in stabilizing the trimeric LUBAC structure.

## Materials and Methods

### Protein structure prediction by AlphaFold2

The amino acid sequences of the proteins HOIP, HOIL-1L, and SHARPIN were obtained from the NCBI website ( www.ncbi.nlm.nih.gov), and the LUBAC protein structure was obtained from the RCSB Protein Data Bank ( www.rcsb.org) (PDB ID: 5Y3T).

The monomeric subunit structures of HOIP-UBA, HOIL-1L-UBL, and SHARPIN-UBL and the LUBAC complex structure were predicted using the monomer and multimer prediction modules of AlphaFold2 (version 2.3.2), respectively. The complete MSA database was used to verify the accuracy of the predictions during the whole process, and optimization was performed on all the predicted models. The selected structures for the experiment were those with the highest predicted quality, *i*. *e*., those with the highest pLDDT scores.

### Alignment of prediction results

Structural alignment between the predicted protein structures and the LUBAC crystal structure was performed using PyMOL 2.5.0 ( www.pymol.org) to further validate the accuracy of the predicted structures. The root mean square deviation (RMSD) between the structures was measured to analyze the relationship between the predicted structures and the crystal structure.

### Analysis of protein-protein interactions

To identify the residues involved in inter-subunit interactions within LUBAC, the changes in residue surface area were analyzed using the interface residues script of PyMOL software. This analysis was conducted on both monomeric and complex structures to identify the residues involved in the interactions. The residues involved in the interactions were visualized using a stick model for better representation. Additionally, protein molecular structures, nonpolar interactions between proteins, and protein surface potentials were visualized.

### Measurement of the binding free energy for single-point mutations

To quantify the impact of single point mutations on protein-protein interactions, the changes in binding free energy (ΔG) upon introducing mutations at the same position in different structures were measured. GeoPPI ( https://github.com/Liuxg16/GeoPPI) was used to measure the effects of single point mutations on interactions in different structures. The corresponding ΔG values were recorded by mutating the residue position selected in GeoPPI to alanine. Finally, a bar graph depicting the inter-subunit interactions in different structures was generated using Origin2021 ( www.originlab.com).

### Molecular dynamics simulation

Molecular dynamics simulations of the HOIL-1L and HOIP complexes were carried out using Gromacs2022.3 software ( http://www.gromacs.org). The obtained potential data were added to the topology file of the molecular dynamics system. The simulations were conducted under static conditions at a temperature of 300 K and atmospheric pressure. The Amber99sb-ildn force field was employed with water molecules as the solvent (using the Tip3p water model), and the simulation system’s total charges were neutralized by adding a certain concentration of Na ^+^ ions. The molecular dynamics simulation system was first subjected to energy minimization using the steepest descent method. Subsequently, 100,000 steps of equilibration were performed under both isothermal-isochoric (NVT) and isothermal-isobaric (NPT) ensembles, with a coupling constant of 0.1 ps and a duration of 100 ps each. Finally, a 5,000,000-step free molecular dynamics simulation was run with a time step of 2 fs, resulting in a total simulation time of 100 ns. After completing the simulation, trajectory analysis was performed using the built-in tools of the software to calculate various data, such as the root-mean-square deviation (RMSD) and root-mean-square fluctuation (RMSF) of amino acid motion trajectories, as well as the protein gyration radius.

### Molecular kinetics experiments

The construction of the target proteins for the expression system was conducted as described previously [ 4, 14] . Briefly, DNA fragments encoding the human HOIL -1 L-UBL domain (residues 55‒135), the HOIL-1L domain (residues 5‒135), the SHARPIN-UBL domain (residues 206‒309), the SHARPIN domain (residues 127‒309), the HOIP-UBA domain (residues 466‒629), the HOIP domain (residues 667‒695), and the HOIP domain (residues 466‒695) were cloned and inserted into the pET SUMO and pET-32a vectors harboring His _6_-SUMO and His _6_-TrxA tags at the N-terminus, respectively. The recombinant proteins were expressed in *E*. *coli* BL21 (DE3) cells at 16°C and purified through affinity chromatography and size-exclusion chromatography to obtain protein samples with a purity of ≥ 90%.

The impact of LTM domains within the LUBAC core on the binding affinity among subunits was investigated by using the Biacore T200 platform, as was the influence of HOIP (629‒695) on the binding affinity between HOIL-1L-UBL and HOIP. Affinity constants (KDs) were calculated by fitting the data using Biacore T200 evaluation software.

The ITC experiments were conducted on the MicroCal PEAQ-ITC platform, and the experimental data were analyzed and processed using MicroCal PEAQ-ITC Analysis Software.

## Results

### Prediction of LUBAC subunit structures by AlphaFold2

**Figure 1:**
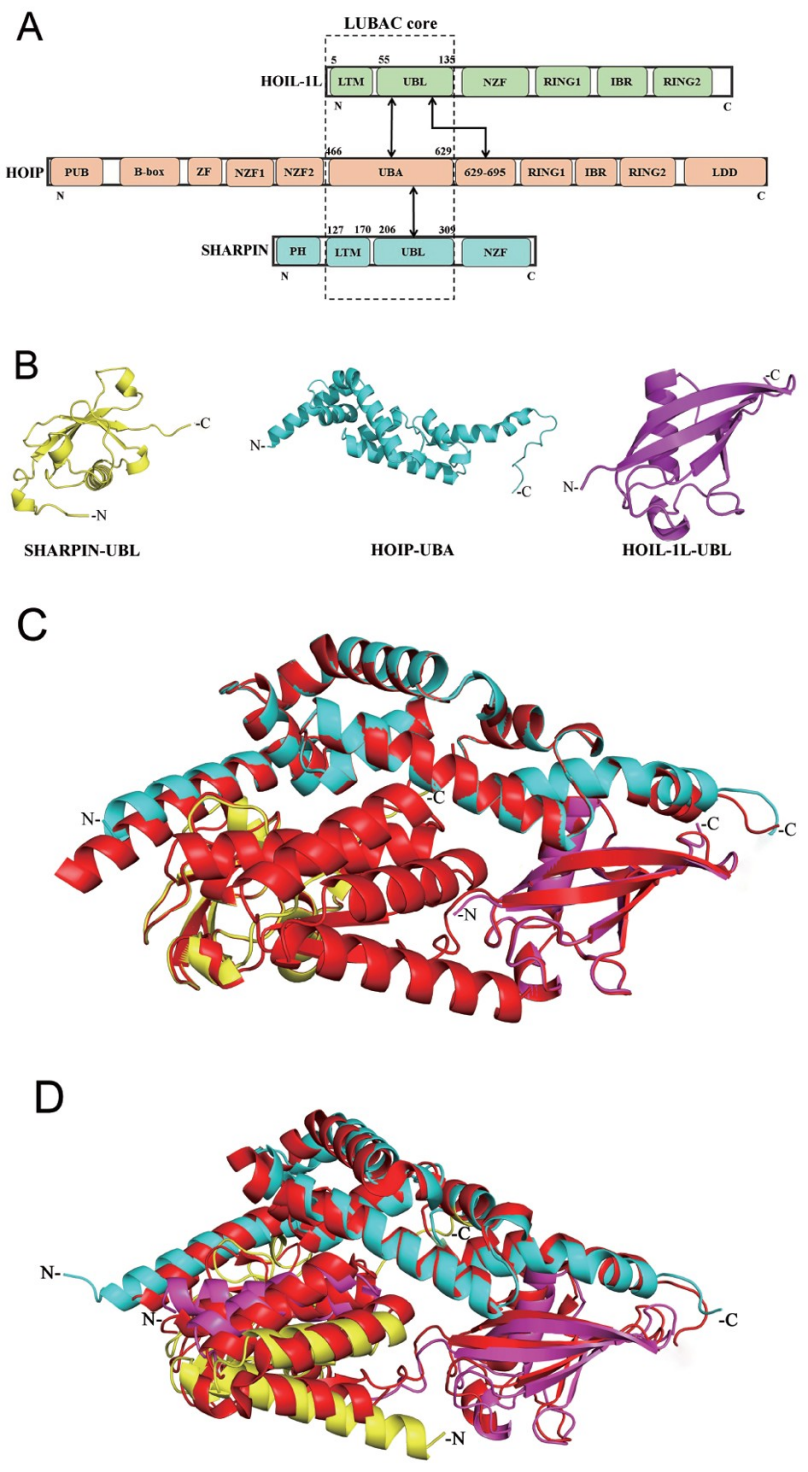
Figure 1 AlphaFold-predicted core domain structures of LUBAC subunits (A) The boundaries of the structural domains of HOIP, HOIL-1L, and SHARPIN in the LUBAC ternary complex are depicted. The LUBAC core domain is indicated by the dashed line. The black arrows represent the interactions between the different structural domains examined in this study. (B) AlphaFold-predicted individual structures of LUBAC subunits. (C) The predicted individual subunits overlapping with 5Y3T. (D) AlphaFold-predicted complex structure of LUBAC overlapping with 5Y3T. HOIP-UBA (cyan), SHARPIN-UBL (yellow), HOIL-1L-UBL (purple), 5Y3T (red).

The locations of the core domains of LUBAC are shown in Figure 1A. LTM motifs and the HOIP (629‒695) domain are presented in the schematic diagram. The residue sequences of three subunits, HOIP-UBA (480‒639), SHARPIN-UBL (206-309), and HOIL-1L-UBL (55‒135), which form the LUBAC core domain, were individually input into the monomer structure prediction module of AlphaFold2 for structure prediction. The results revealed that HOIP-UBA exhibited an irregular elongated shape composed of 7 α-helices of varying lengths stacked together. The structures of SHARPIN-UBL and HOIL-1L-UBL were similar, with 4 β-folds of different lengths surrounding a central α-helix, which jointly formed a structure that resembled an hourglass shape, with larger ends and a smaller middle chamber ( Figure 1B). Then, the predicted structures were compared to the crystal structure of the LUBAC core domain (PDB ID: 5Y3T) through structural alignment. The RMSD of the predicted HOIP-UBA structure (pLDDT ≥ 95) compared to that of 5Y3T was 0.380 Å, that of HOIL-1L-UBL (pLDDT ≥ 85) was 0.407 Å, and that of SHARPIN-UBL (pLDDT ≥ 85) was 0.272 Å ( Figure 1C). Using the complex structure prediction module of AlphaFold2, the trimeric structure formed by the catalytic subunit HOIP-UBA and the accessory subunits HOIL-1L-UBL and SHARPIN-UBL was predicted and analyzed (pLDDT ≥ 95, DockQ ≥ 0.90). Compared to the experimental result for 5Y3T, the predicted structure of AlphaFold2 accurately depicted the correct folding pattern between the three subunits ( Figure 1D). Therefore, the use of AlphaFold2 for predicting the structure of the LUBAC core domain was successful.

### Removal of the LTM motif influences the HOIP-UBA conformation

**Figure 2:**
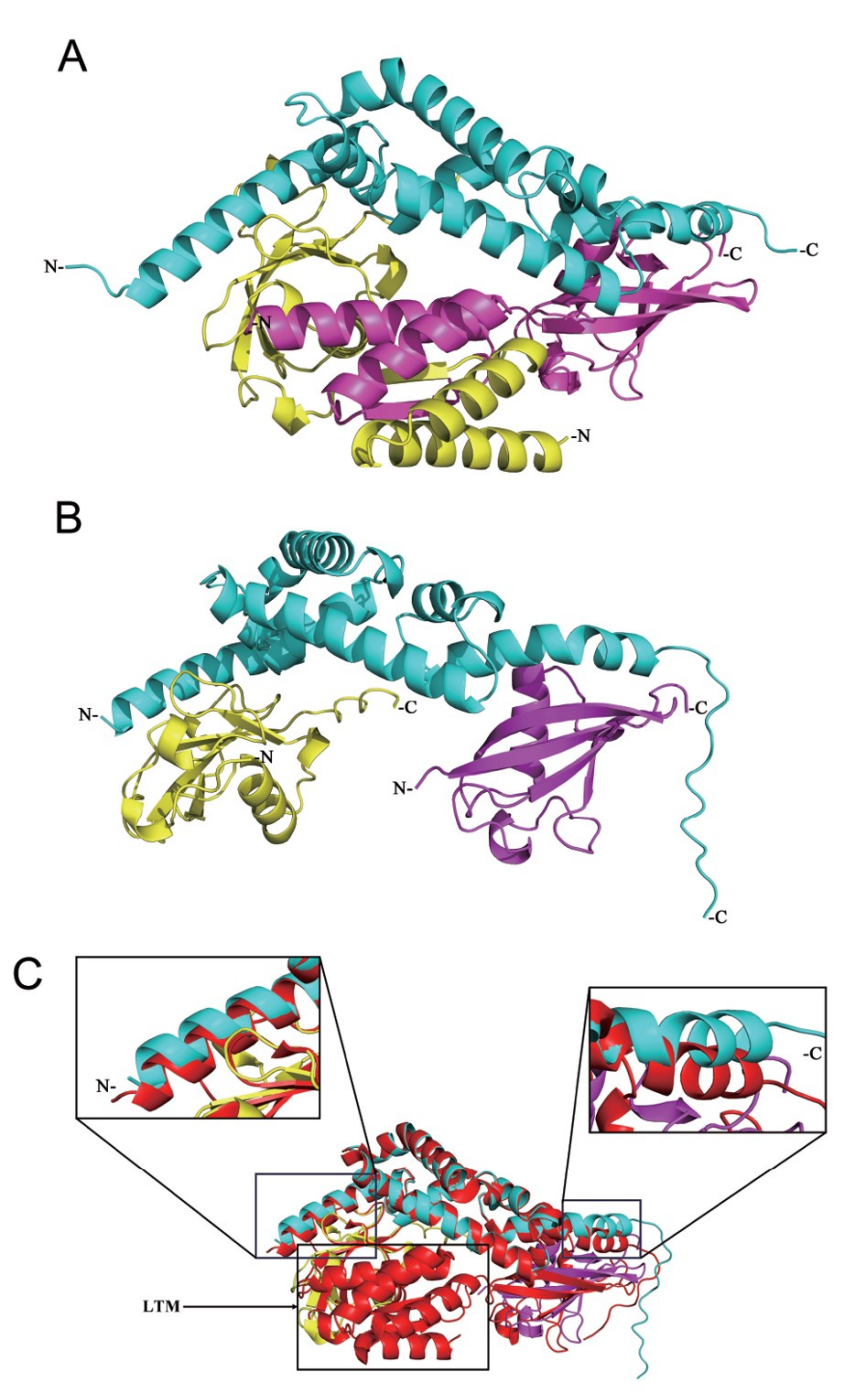
Figure 2 Comparison of two LUBAC structures predicted by AlphaFold2 (A) Predicted structure of LUBAC with the LTM motif. (B) Predicted structure of LUBAC without the LTM motif. (C) Comparison of two LUBAC structures by superimposing (A) to (B). The structure with the LTM motif is colored red. HOIP-UBA (cyan), SHARPIN-UBL (yellow), and HOIL-1L-UBL (purple).

The LTM motif is formed by the co-folding of dual α-helices from SHARPIN-UBL and the N-terminal region of HOIL-1L-UBL. In the trimeric structure of LUBAC, the LTM motif, composed of 4 α-helices stacked together, is positioned on the outer side, without direct contact with the HOIP-UBA structural domain and at a considerable distance. AlphaFold2 successfully predicted that the trimeric structure of LUBAC contained an LTM motif (pLDDT ≥ 95, DockQ ≥ 0.90) ( Figure 2A). The LTM motif is located between SHARPIN-UBL and HOIL-1L-UBL. Furthermore, experimental AlphaFold2 prediction generated the LUBAC structure without the LTM motif (pLDDT ≥ 95, DockQ ≥ 0.95) ( Figure 2B). Comparing Figure 2A to Figure 2B, the positions of the 3 subunits in both structures are identical, validating the accuracy of the predictions. Further investigation of the impact of LTM on the overall structure, especially on HOIP-UBA, was conducted using PyMOL software. By superimposing the predicted structures shown in Figure 2A,B, it was surprising to observe that SHARPIN-UBL and HOIL-1L-UBL perfectly overlapped. However, significant deviations were observed at the interacting regions between the terminal ends of the HOIP-UBA structure and the SHARPIN-UBL and HOIL-1L-UBL domains ( Figure 2C). Therefore, the conformational changes in HOIP-UBA within the trimeric structure are influenced by the LTM motif.

### Effects of the LTM motif on the interactions among LUBAC subunits

Within the trimeric structure of LUBAC, LTM indirectly influences the conformational changes in the HOIP-UBA subunit. The N- and C-terminal regions of HOIP-UBA are involved in interactions with SHARPIN-UBL and HOIL-1L-UBL. LTM may induce structural changes in HOIP-UBA by altering the inter-subunit interactions within LUBAC. GeoPPI is capable of predicting the impacts of mutations on binding affinity (ΔG), which reflects protein-protein interactions. Therefore, the experimental approach employed GeoPPI to calculate the variations in protein-protein interactions. Based on the predicted structure of LUBAC without the LTM motif, PyMOL software was used to analyze all possible residues involved in the interaction between the catalytic subunit HOIP and the accessory subunit HOIL-1L-UBL. GeoPPI was then employed to predict the effects of amino acid mutations on the binding affinity of the subunits.

**Figure 3:**
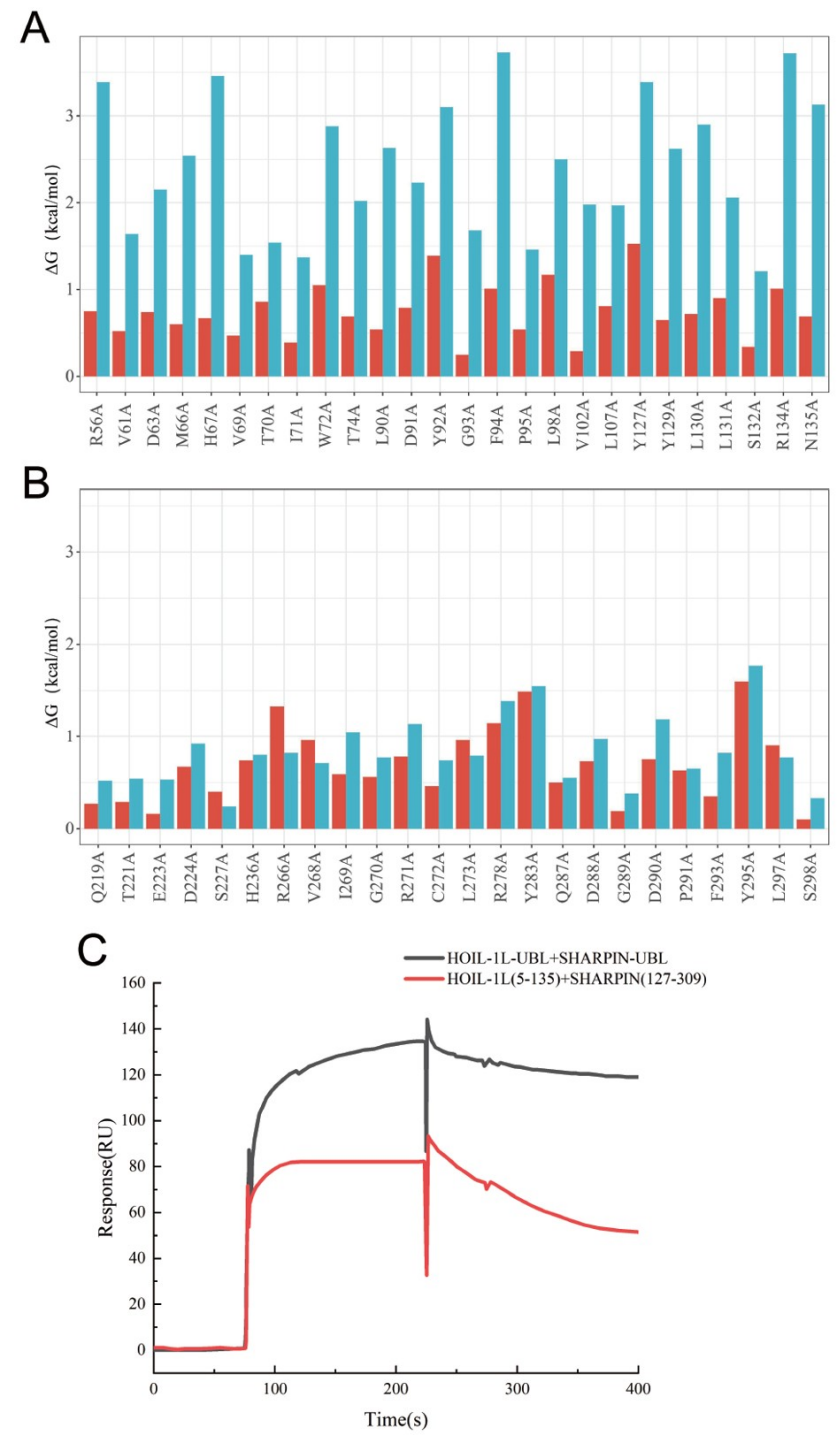
Figure 3 Effects of LTM domains on inter-subunit interactions within the LUBAC core domain (A) ΔG values of HOIL-1L-UBL mutants compared to those of HOIP-UBA. The X-axis represents the mutated residues of HOIL-1L-UBL. (B) ΔG values of SHARPIN-UBL mutants. The X-axis represents the mutation residues of SHARPIN-UBL. The red bar graph represents the ΔG values for mutations in the LUBAC structure (5Y3T) containing the LTM motif, while the blue bar graph represents the ΔG values for mutations in the predicted structure of LUBAC without the LTM motif. (C) The binding affinity between SHARPIN and HOIL-1L with HOIP-UBA (466‒630) was analyzed using SPR.

In the presence of LTM in 5Y3T, any residue mutation to alanine within the interface of the catalytic subunits HOIP-UBA and HOIL-1L-UBL led to significant changes in the binding free energy. As a comparison, the mutations were performed on the same residues of HOIL-1L-UBL in the predicted structures without LTM. Surprisingly, the ΔG values generated by these mutations were significantly greater than those generated by 5Y3T ( Figure 3A). A similar measurement was conducted to assess the Gibbs free energy changes resulted from single-point mutations between SHARPIN-UBL and HOIP-UBA. Compared to those of 5Y3T, only mutations in S227, R266, L273, and L297 of SHARPIN-UBL exhibited larger ΔG values than those predicted in the absence of LTM ( Figure 3B). Overall, LTM reduced the ΔG values of amino acid mutations within SHARPIN-UBL, similar to what was observed in HOIL-1L-UBL. LTM decreased the binding free energy changes caused by single-point mutations, thereby reducing the interplay between the core subunits of LUBAC.

Then, we used SPR assays to verify the computational results mentioned above. Purified and diluted HOIL-1L (5‒135) and SHARPIN (127‒309) at the same concentration were mixed and incubated at 16°C for 2 h before SPR analysis to examine their interaction with HOIP-UBA (466‒630). For comparison, HOIL-1L-UBL and SHARPIN-UBL without the LTM domain were analyzed in a similar manner. The SPR results indicated that the KD for the interaction between HOIL-1L-UBL and SHARPIN-UBL with HOIP-UBA was (7.21±0.22)×10 ^‒8^ M [Rmax (RU=129.83), Chi ^2^ (RU ^2^)= 0.214]. The KD value between HOIL-1L (5‒135) and SHARPIN (127‒309), which contain the LTM domain with HOIP-UBA, was (4.96±0.31)×10 ^‒7^ M [Rmax (RU=82.31), Chi ^2^ (RU ^2^)=0.125]. These results demonstrated that the presence of the LTM domains of HOIL-1L (5‒135) and SHARPIN (127‒309) reduced the binding of the catalytic subunit HOIP-UBA to the core structural domains (UBL) of the two auxiliary subunits ( Figure 3C).

### Interactions between HOIL-1L-UBL and HOIP (629‒695) in LUBAC

**Figure 4:**
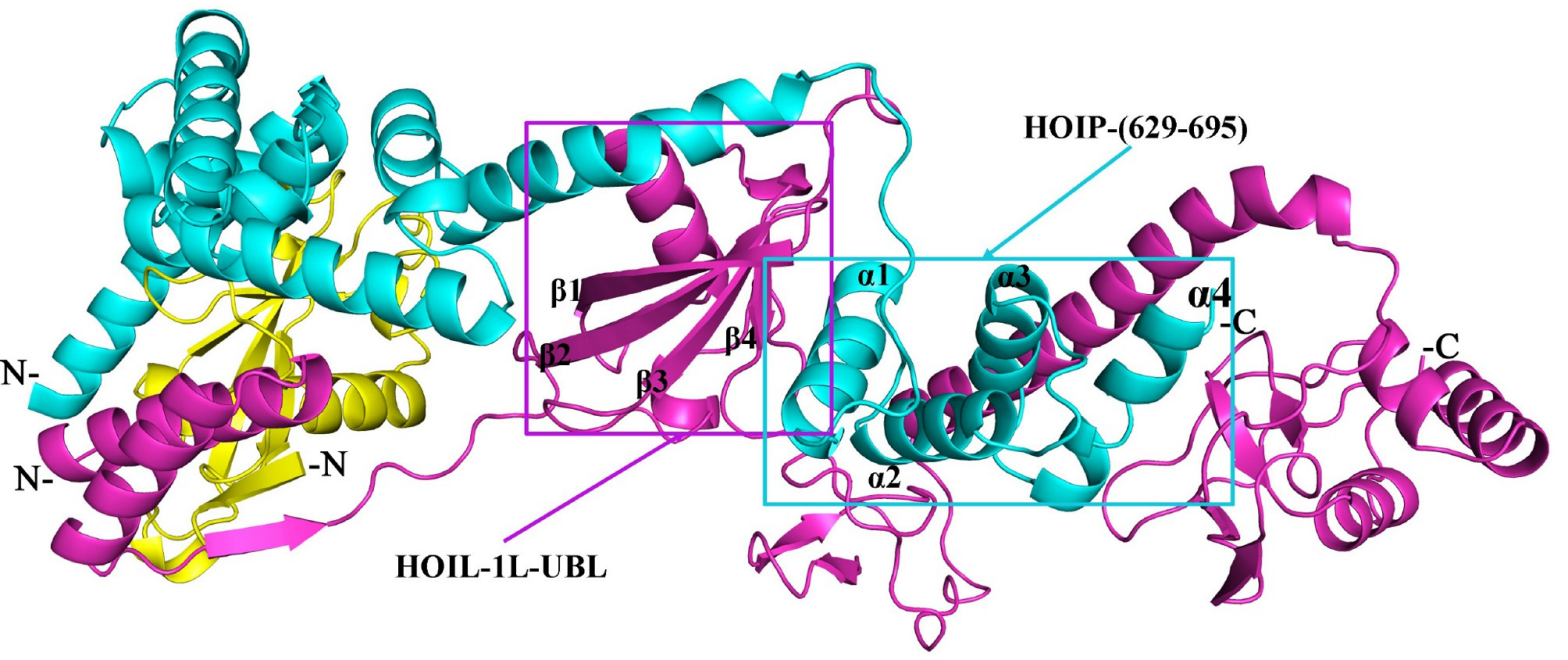
Figure 4 The structure of HOIP (625‒695) in LUBAC HOIP (cyan), SHARPIN (yellow), and HOIL-1L (purple). The positions of HOIL-1L-UBL and HOIP (629‒695) in the complex structure are indicated by purple and cyan rectangles, respectively.

The complex structure formed by the interaction between HOIL-1L-UBL and HOIP (629‒695) in LUBAC was successfully predicted by AlphaFold2 (pLDDT ≥ 80, DockQ ≥ 0.8). In this structure, we focused on HOIP (625‒695), which is located at the C-terminus of HOIP-UBA, lies between HOIP-UBA and HOIP-RBR and adopts an extended 4-helix bundle conformation ( Figure 4). The 4 α-helices within this structure are arranged at different angles and are approximately equal in length. Similar to the binding mode of HOIP-UBA and HOIL-1L-UBL, the α1 helix of HOIP (625‒695) forms a tight grip with the UBL domain of HOIL-1L through its 4-stranded β-sheet fold. Overall, the α-helix at the C-terminus of HOIP-UBA and the α1 helix at the N-terminus of HOIP (625‒695) possess an inverted V shape and form a sandwich model with the HOIL-1L-UBL domain to further maintain the structural stability of LUBAC.

### Interaction interface between HOIL-1L-UBL and HOIP (629‒695)

**Figure 5:**
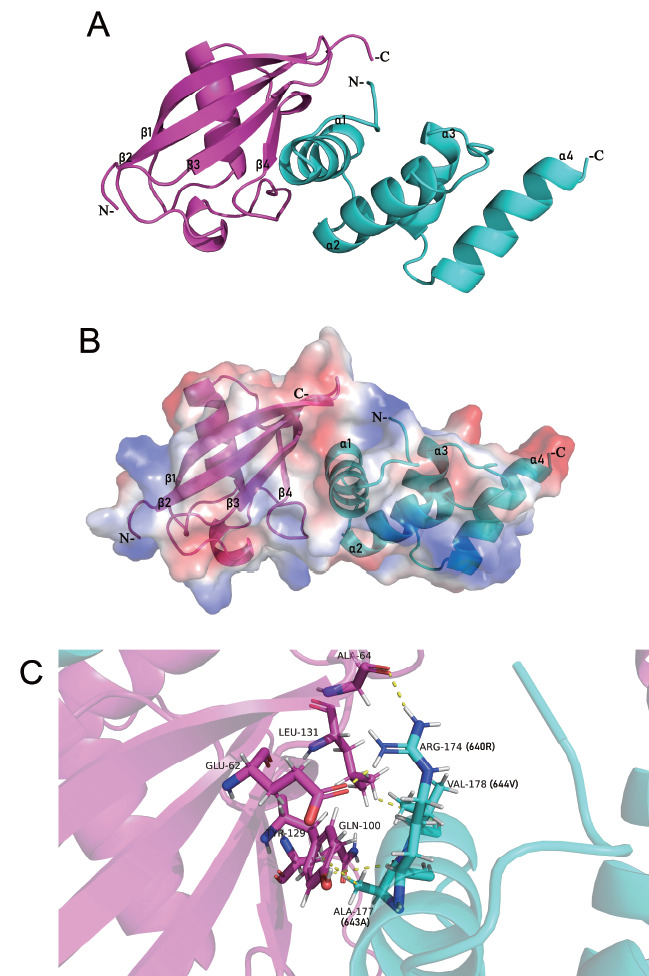
Figure 5 The interface between HOIL-1L-UBL and HOIP (629‒695) HOIP-UBA (cyan) and HOIL-1L-UBL (purple). (A) Cartoon representation of the complex formed by HOIL-1L-UBL and HOIP (629‒695). (B) Surface potential distribution of HOIL-1L-UBL and HOIP (629‒695). Red represents negative potential. Blue represents positive potential. (C) Analysis of the interaction interface between HOIL-1L-UBL and HOIP (629‒695).

Based on the above predicted structure, a detailed structural analysis was performed. The results revealed a tight binding interaction between the HOIL-1L-UBL domain and the surface of the HOIP (629‒695) domain ( Figure 5A). Potential energy analysis of the complex surface indicated that the interaction between the HOIL-1L-UBL domain and HOIP (629‒695) is mediated by a combination of electrostatic forces, hydrogen bonding, and hydrophobic interactions. The UBL domain of HOIL-1L forms a pocket with a negative electrostatic potential and harbors 4 β-strands. The positive potential on the surface of the HOIP (629‒695) α1 helix interacts with the negatively charged pocket of the HOIL-1L UBL domain, resulting in a significant potential difference on the complex surface ( Figure 5B). In addition to electrostatic potential, the interaction between residues contributes to structural stability. Specifically, residues E62, A64, Q100, Y129, and Q131 from HOIL-1L, along with residues R640, 643A, and V644 from HOIP, jointly enhance LUBAC stability ( Figure 5C).

### HOIP (629‒695) enhances the interaction between HOIP-UBA and HOIL-1L-UBL

In the complex structure of LUBAC ( Figure 4), the spatial arrangement of the HOIP-UBA and HOIP (629‒695) domains results in an inverted V-shaped structure. The HOIL-1L-UBL structure is sandwiched between layers, creating a sandwich-like configuration. HOIP-UBA and HOIP (629‒695) are continuous domains within HOIP and are positioned close to each other. As HOIP-UBA and HOIP (629‒695) are located on opposite sides of HOIL-1L-UBL, the presence of HOIP (629‒695) may induce changes in the interaction between HOIP-UBA and HOIL-1L-UBL.

**Figure 6:**
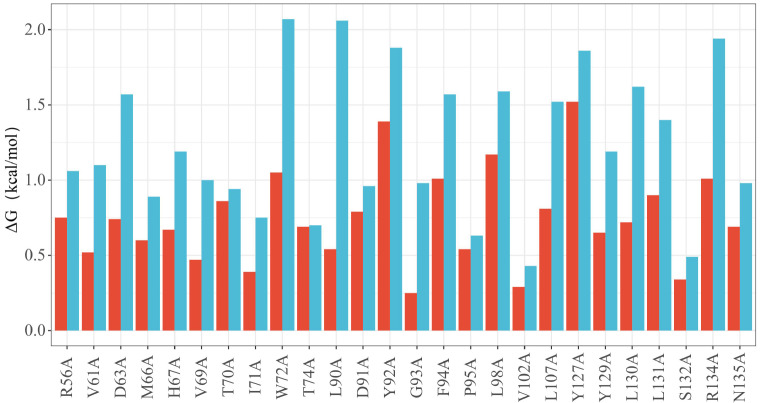
Figure 6 Influence of single-point mutations on the ΔG values The red bar chart represents the mutants of LUBAC without the HOIP (629‒695) domain (5Y3T). The blue bar chart represents the mutants in the predicted LUBAC structure with the HOIP (629‒695) domain. The X-axis represents the mutated residues of HOIL-1L-UBL.

To investigate the impacts of the HOIP (629‒695) domain in LUBAC on the interaction between HOIP-UBA and HOIL-1L-UBL, the binding affinity between the structural domains was calculated using GeoPPI software. The results showed that the presence of the HOIP (629-695) domain resulted in a significant increase in ΔG for single-point mutations of HOIL-1L-UBL compared to that of HOIP-UBA and further to that of 5Y3T. Thus, HOIP (629‒695) enhances the interaction between HOIP-UBA and HOIL-1L-UBL in LUBAC ( Figure 6).

### The formation of a stable complex between HOIP (466‒695) and HOIL-1L-UBL

The computational results from GeoPPI demonstrated that HOIP (629‒695) enhances the interaction between HOIP-UBA and HOIL-1L-UBL in LUBAC. Gromacs, a widely used and powerful molecular dynamics simulation software, was employed to investigate the structural stability of the complex formed by the relative HOIP and HOIL-1L domains. Molecular dynamics simulations were performed on the extended structures of HOIP UBA from residues 466‒695 and on HOIL-1L-UBL, which were compared to those containing only the HOIP (629‒695) domain, to explore the contribution of the side region of HOIP UBA to complex stability.

**Figure 7:**
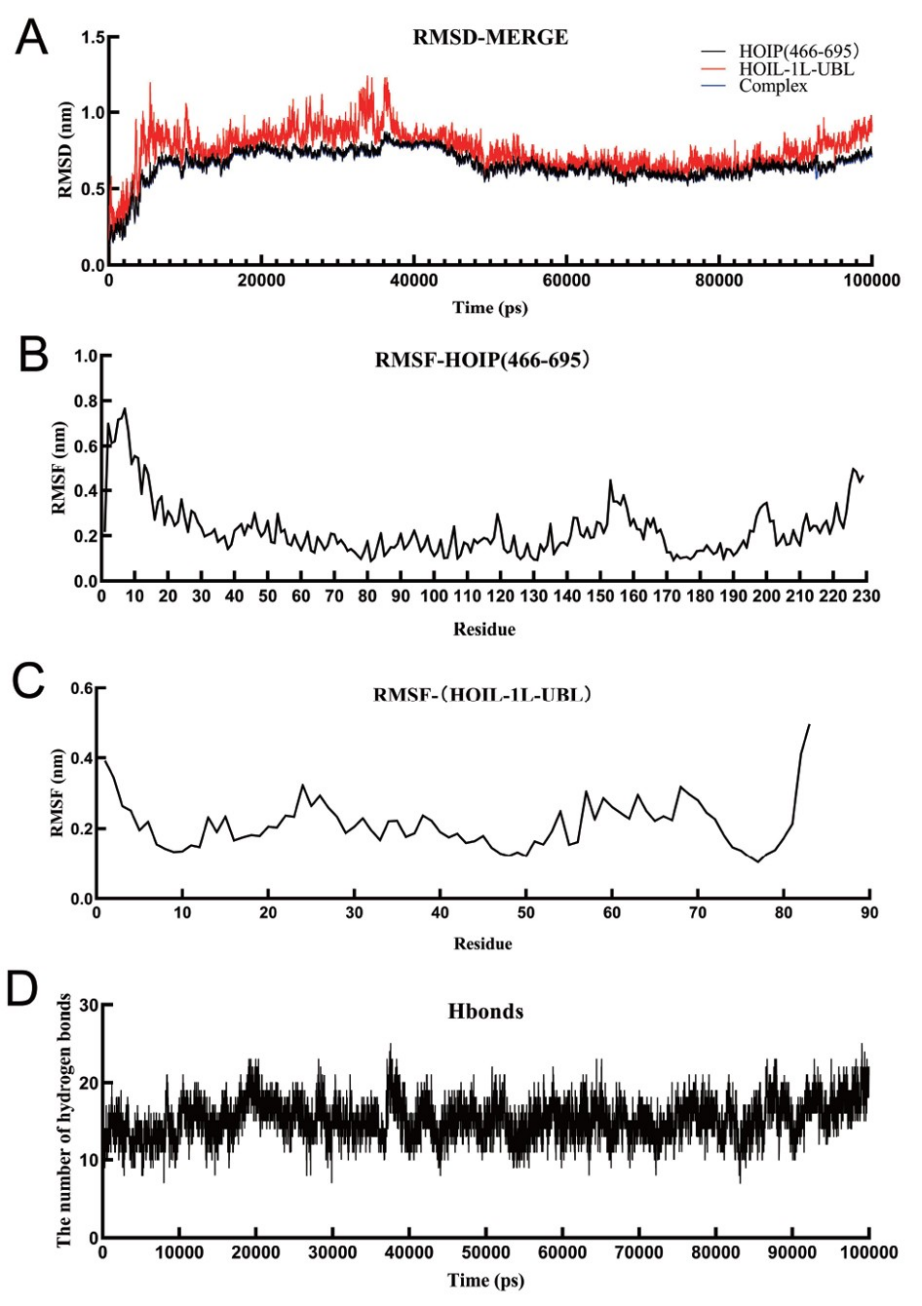
Figure 7 Gromacs simulation results of the complex formed by HOIP (466‒695) and HOIL-1L-UBL (A) RMSD variation curve of the target protein over the Gromacs simulation time. (B) RMSF variation curve of different amino acids in HOIP (466‒695). (C) RMSF variation curve of different amino acids in HOIL-1L-UBL. (D) Hydrogen bond variation curve of the target protein over the Gromacs simulation time.

The RMSD results indicated that the protein structures of HOIP (466‒695) and HOIL-1L-UBL remained relatively stable throughout the simulation, with an overall trend of initial increase followed by subsequent decrease, and the overall RMSD values were below 1 nm. The RMSD trend of the complex was consistent with that of HOIP (466‒695). These findings suggest that the interaction between HOIP (466‒695) and HOIL-1L-UBL has a minimal impact on the overall protein structure ( Figure 7A).

RMSF can characterize the flexibility and degree of amino acid motion in a protein throughout the simulation process. The RMSF values of both the HOIP (466‒695) and HOIL-1L-UBL models remained relatively stable during the simulation, with overall amplitudes maintained at low levels. The majority of the HOIP (466‒695) proteins exhibited RMSF values ranging from 0.15 nm to 0.40 nm throughout the simulation ( Figure 7B), while different regions of HOIL-1L-UBL maintained stable RMSF values of approximately 0.20 nm ( Figure 7C). This suggests that the interaction between HOIP (466‒695) and HOIL-1L-UBL has little effect on the stability of the internal protein structure.

The number of hydrogen bonds between HOIP (466‒695) and HOIL-1L-UBL remained relatively stable during the simulation. The number of hydrogen bonds between HOIP (466‒695) and HOIL-1L-UBL ranged from 10 to 20 throughout the simulation ( Figure 7D). These results indicated favorable interaction characteristics and stability between HOIP (466‒695) and HOIL-1L-UBL. The simulation results demonstrated the formation of a structurally stable complex between HOIL-1L-UBL and the HOIP (466‒695) domain.

### Evaluation of binding kinetics

**Figure 8:**
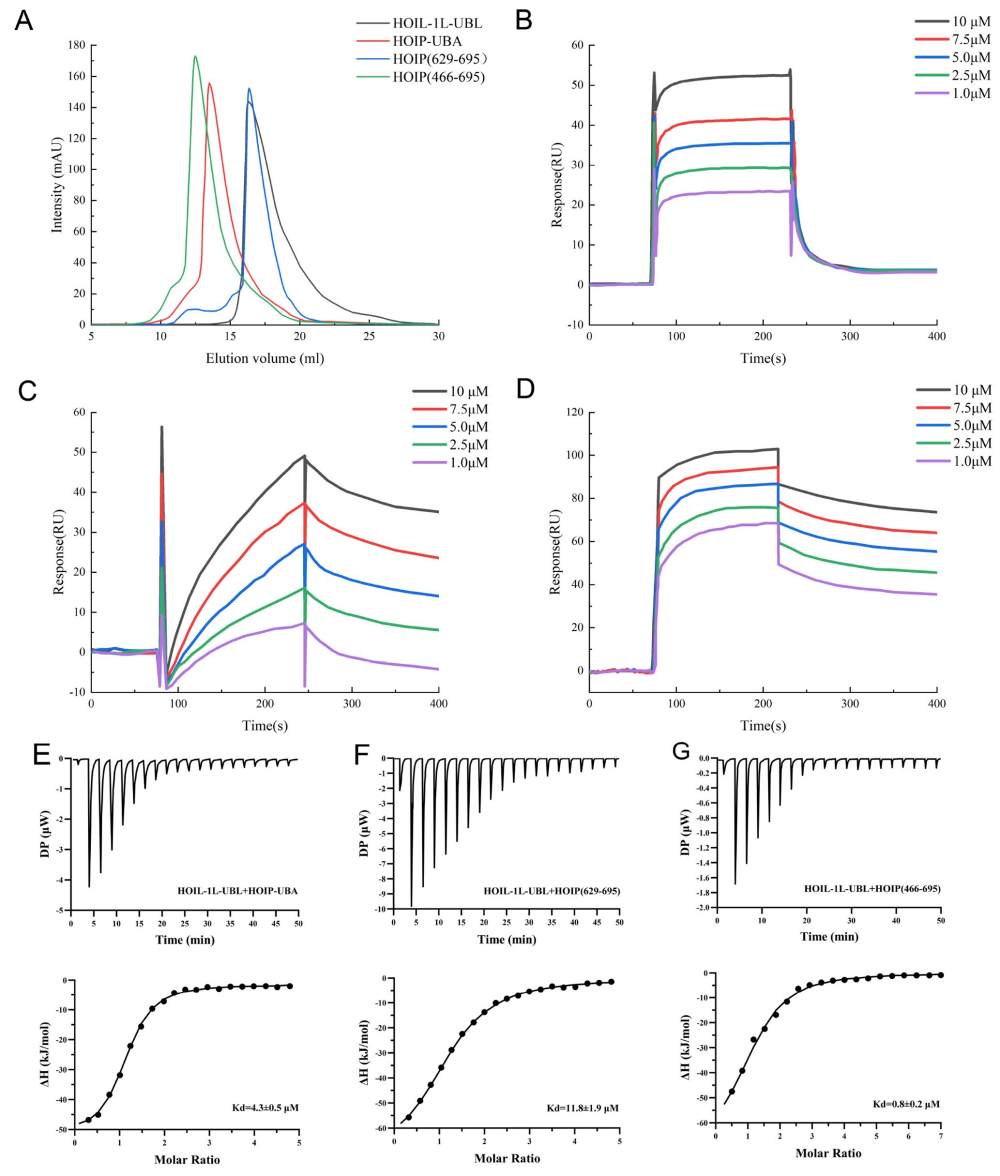
Figure 8 Interactions between HOIL-1L-UBL and HOIP were analyzed by SPR (A) Gel filtration chromatogram of the purified target protein. (B) Evaluation of the binding affinity between HOIL-1L-UBL and HOIP-UBA by immobilizing HOIL-1L-UBL on the SPR sensor chip. Rmax (RU)=53.95, Chi 2 (RU 2)=2.432. (C) Evaluation of the binding affinity between HOIL-1L-UBL and HOIP (629‒695) by immobilizing HOIL-1L-UBL on the SPR sensor chip. Rmax (RU)=56.35, Chi 2 (RU 2)=1.174. (D) Evaluation of the binding affinity between HOIL-1L-UBL and HOIP (466‒695) by immobilizing HOIL-1L-UBL on the SPR sensor chip. Rmax (RU)=101.87, Chi 2 (RU 2)=0.103. (E) ITC measurements of the binding affinity between HOIL-1L-UBL and HOIP-UBA. (F) ITC measurements of the binding affinity between HOIL-1L-UBL and HOIP (629‒695). (G) ITC measurements of the binding affinity between HOIL-1L-UBL and HOIP (466‒695).

Structural analysis and molecular dynamics simulations of the trimeric LUBAC predicted an increased interaction between HOIP (466‒695) and HOIL-1L-UBL, which enhanced the structural stability between HOIP and HOIL-1L. To further analyze the binding affinity between HOIL-1L-UBL and HOIP, samples of 4 target proteins, HOIL-1L-UBL, HOIP-UBA, HOIP (629‒695), and HOIP (466‒695), were prepared for SPR experiments. After purification by affinity chromatography and gel filtration chromatography, high-purity samples were obtained ( Figure 8A). Kinetic studies were conducted to investigate the interaction between HOIL-1L-UBL and three HOIP domains of different lengths.

The SPR results demonstrated different interactions between HOIL-UBL and HOIP-UBA, HOIP (629‒695), and HOIP (466‒695) ( Figure 8B‒D). Further analysis of the SPR curves revealed that the binding and dissociation of HOIP-UBA with HOIL-1L-UBL were relatively rapid ( Figure 8B). In contrast, the binding and dissociation of HOIP (629‒695) with HOIL-1L-UBL were slower ( Figure 8C). To further analyze the effect of HOIP (629‒695) on the interaction between HOIP and HOIL-1L-UBL, the data were fitted and analyzed using Biacore T200 software. The KD value between HOIP (629‒695) and HOIL-1L-UBL was (9.89±0.31)×10 ^‒7^ M, and the KD value between HOIP-UBA and HOIL-1L-UBL was (5.43±0.68)×10 ^‒7^ M in the absence of the HOIP (629‒695) structure. When HOIP (629‒695) was combined with HOIP-UBA, the KD value between HOIP (466‒695) and HOIL-1L-UBL was (3.59±0.43)×10 ^‒7^ M. Furthermore, ITC assays indicated that the affinity between HOIP (466‒695) and HOIL-1L-UBL (KD value: 0.8±0.2 μM) was significantly greater than that between HOIP-UBA and HOIL-1L-UBL (KD value: 4.3±0.5 μM) ( Figure 8E‒G). These results indicated that the domain of HOIP (629‒695) enhances the binding between HOIP and HOIL-1L-UBL.

## Discussion

This study computationally analyzed the partial and trimeric structures of the linear ubiquitin chain assembly complex (LUBAC) to explore the influences of different components on trimeric structure stability. AlphaFold2 was used to predict the trimeric structures of the LUBAC core domain, which were compared with the experimental crystal structure (PDB ID: 5Y3T). The RMSD values of the monomers HOIP-UBA, HOIL-1L-UBL, and SHARPIN-UBL concerning the crystal structure 5Y3T were all less than 0.5 Å. These results demonstrated the successful prediction of the aforementioned monomeric structures by AlphaFold2. Subsequently, two types of LUBAC trimeric structures, one without the LTM motif and the other with the LTM motif, were predicted using AlphaFold2, and the results were compared with the crystal structure of 5Y3T. In both types of LUBAC core domain structures, the RMSD values of the HOIP-UBA, HOIL-1L-UBL, or SHARPIN-UBL subunit compared to the LUBAC core domain (PDB ID: 5Y3T) were all less than 1 Å. The predicted parameters for the trimeric LUBAC structure without the LTM motif were pLDDT ≥ 95 and DockQ ≥ 0.95, while the parameters for those with the LTM motif were pLDDT ≥ 95 and DockQ ≥ 0.9. Therefore, both types of LUBAC trimeric structures were considered to be within the error range of the structure 5Y3T, indicating that this deep learning approach can be used for subsequent experiments to obtain the correct prediction results.

The predicted structures of various components of LUBAC were analyzed using PyMOL software. The structural alignment results indicated that the two ends of the HOIP-UBA structures from the LUBAC without and with the LTM motif could not overlap completely. It has been previously verified that the HOIP-UBA domain undergoes conformational changes during the trimeric binding process [7]. In LUBAC, the LTM structure does not directly interact with HOIP-UBA but indirectly affects the conformational changes in HOIP-UBA through HOIL-1L-UBL and SHARPIN-UBL.

To investigate the impacts of LTM on the interactions among LUBAC structures, a novel framework called GeoPPI, which utilizes a geometric representation learned from protein structures, was employed to simulate the effects of mutations on binding affinity accurately. The binding free energy (ΔG) caused by single amino acid mutations between the predicted structure of the LUBAC core domain without the LTM motif and the experimental structure 5Y3T harboring the LTM motif was measured through GeoPPI. In LUBAC, specific amino acid mutations were identified at positions where HOIL-1L-UBL and SHARPIN-UBL directly interact with HOIP-UBA, and ΔG values were measured. Compared to those in the 5Y3T structure, the ΔG values in LUBAC without the LTM motif were consistently greater after the corresponding amino acid was mutated to alanine. This effect was particularly pronounced in the HOIL-1L-UBL group. The results indicated that the LTM domain reduces the ΔG associated with single-point mutations in LUBAC, leading to a reduction in the binding affinity between HOIP-UBA and HOIL-1L-UBL or SHARPIN-UBL within the LUBAC complex. However, the SPR results support this observation, and the influence of the LTM domain on the overall structural stability of LUBAC remains uncertain. This finding further supports the essential role of the LTM motif, which is a synergistic structural domain found in HOIL-1L and SHARPIN [11].

In addition to the core domain of LUBAC, AlphaFold2 successfully predicted larger structures of LUBAC. In this structure, the HOIP-UBA structure adjacent to the C-terminus of HOIP (629‒695) adopts a unique 4 α-helix bundle conformation. The first α-helix of the N-terminus of HOIP (629‒695) is closely connected to HOIL-1L-UBL. HOIP (629‒695) and HOIP-UBA are arranged in an inverted V shape, with the HOIL-1L-UBL structure sandwiched between them. Structural analysis revealed the stable binding of HOIP (629‒695) with HOIL-1L-UBL. The impacts of single point mutations on the interaction between HOIP and HOIL-1L were calculated in the presence of the HOIP (629‒695) structure using GeoPPI. Compared to 5Y3T, the single point mutations were found to enhance the binding affinity between HOIP-UBA and HOIL-1L-UBL, indicating that the interaction between HOIP (629‒695) and HOIL-1L-UBL enhances the structural stability between HOIP-UBA and HOIL-1L-UBL.

Gromacs software is a commonly used molecular dynamics simulation tool that can be used to simulate the dynamic processes of protein-protein interactions and evaluate various indicators. Here, we investigated the stability of the protein structures formed by mutual interactions among HOIP (629‒695), HOIP-UBA, and HOIL-1L-UBL. To align with the subunit structures in LUBAC, HOIP-UBA and HOIP (629‒695) were merged into HOIP (466‒695). Based on the structure of the complex of HOIP (466‒695) and HOIL-1L-UBL, a 100-ns simulation of the complex was performed using Gromacs. The simulation results showed that the RMSD values of HOIP (466‒695) and HOIL-1L-UBL remained low during the simulation period, indicating greater structural stability of the target protein.

To evaluate the protein dynamics, the indicator RMSF was used to calculate the average atomic position to indicate the flexibility of the protein. In this simulation, we calculated the RMSF values of the HOIP (466‒695) and HOIL-1L-UBL proteins and generated RMSF plots. The results showed that the RMSF values of the target proteins remained stable throughout the simulation, and there were no significant impacts on the stability of their internal structures. The number of hydrogen bonds also remained stable during the simulation, maintaining a consistent trend.

SPR and ITC further demonstrated the binding affinity between HOIP and HOIL-1L-UBL. The results showed that the HOIP (466‒695) structure, which contains the HOIP (629‒695) and HOIP-UBA domains, maintains the greater stability of the trimeric LUBAC structure through its interaction with HOIL-1L-UBL. Overall, this study revealed that the presence of the LTM motif in LUBAC decreased the interactions among the subunit domains of SHARPIN-UBL, HOIL-1L-UBL, and HOIP-UBA, thereby reducing the structural stability of LUBAC. However, a distinct impact was observed for HOIP (629‒695) within LUBAC. In the HOIP structure, the UBA region and the HOIP (629‒695) segment together form the HOIP (466‒695) domain, which tightly binds to HOIL-1L-UBL. HOIP (629‒695) enhances the interaction between HOIP-UBA and HOIL-1L-UBL in LUBAC, thus jointly maintaining the structural stability of LUBAC.

In this study, the structures of LUBAC proteins with different subunit lengths were predicted using AlphaFold2. The interactions between the HOIP and UBL domains of SHARPIN in LUBAC were evaluated using GeoPPI. The impacts of the LUBAC LTM motif on the interactions between HOIP and the UBL domains of SHARPIN and HOIL-1L were elucidated. It was concluded that HOIP-UBA interacts with HOIL-1L-UBL in conjunction with HOIP (629‒695). The GeoPPI results demonstrated that HOIP (629‒695) enhances the interaction between HOIP-UBA and HOIL-1L-UBL, thereby increasing the structural stability of LUBAC. The computational analysis revealed that the interaction between the LTM motifs of HOIL-1L and SHARPIN in LUBAC weakens the effects of single-point mutations on LUBAC structural stability. Furthermore, the Gromacs software simulation results generated evidence of significant interactions and structural stability characteristics between HOIP-UBA and HOIP (629‒695) in complex with HOIL-1L-UBL. Finally, the above findings were further verified by SPR molecular dynamics and ITC experiments. Our results provide a novel structural interpretation of the current LUBAC structure and a new approach for further structural and interaction experiments.

## References

[1] Pickart CM, Eddins MJ (2004). Ubiquitin: Structures, functions, mechanisms. Biochim Biophys Acta.

[2] Johnson JAK, Sumner I (2022). On the possibility that bond strain is the mechanism of ring e3 activation in the E2-catalyzed ubiquitination reaction. J Chem Inf Model.

[3] Hicke L, Schubert HL, Hill CP (2005). Ubiquitin-binding domains. Nat Rev Mol Cell Biol.

[4] Ma W, Lu Y, Zuo Y, Wang C, Liu J (2022). Effects of removing a highly conserved disulfide bond in ubiquitin-associated domain of human HOIP on biochemical characteristics. Protein Expr Purif.

[5] Rodriguez Carvajal A, Grishkovskaya I, Gomez Diaz C, Vogel A, Sonn-Segev A, Kushwah MS, Schodl K (2021). The linear ubiquitin chain assembly complex (LUBAC) generates heterotypic ubiquitin chains. Elife.

[6] Peltzer N, Rieser E, Taraborrelli L, Draber P, Darding M, Pernaute B, Shimizu Y (2014). HOIP deficiency causes embryonic lethality by aberrant tnfr1-mediated endothelial cell death. Cell Rep.

[7] Smit JJ, Monteferrario D, Noordermeer SM, van Dijk WJ, van der Reijden BA, Sixma TK (2012). The E3 ligase HOIP specifies linear ubiquitin chain assembly through its RING-IBR-RING domain and the unique LDD extension. EMBO J.

[8] Kausas M, Esposito D, Rittinger K, Fraternali F (2022). Characterisation of HOIP RBR E3 ligase conformational dynamics using integrative modelling. Sci Rep.

[9] Stieglitz B, Rana RR, Koliopoulos MG, Morris-Davies AC, Schaeffer V, Christodoulou E, Howell S (2013). Structural basis for ligase-specific conjugation of linear ubiquitin chains by HOIP. Nature.

[10] Stieglitz B, Morris‐Davies AC, Koliopoulos MG, Christodoulou E, Rittinger K (2012). LUBAC synthesizes linear ubiquitin chains via a thioester intermediate. EMBO Rep.

[11] Liu J, Wang Y, Gong Y, Fu T, Hu S, Zhou Z, Pan L (2017). Structural insights into sharpin-mediated activation of hoip for the linear ubiquitin chain assembly. Cell Rep.

[12] Shimizu S, Fujita H, Sasaki Y, Tsuruyama T, Fukuda K, Iwai K (2016). Differential involvement of the Npl zinc finger domains of sharpin and HOIL-1l in linear ubiquitin chain assembly complex-mediated cell death protection. Mol Cell Biol.

[13] Sato Y, Fujita H, Yoshikawa A, Yamashita M, Yamagata A, Kaiser SE, Iwai K (2011). Specific recognition of linear ubiquitin chains by the Npl4 zinc finger (NZF) domain of the HOIL-1L subunit of the linear ubiquitin chain assembly complex. Proc Natl Acad Sci USA.

[14] Ma W, Lu Y, Qi J, Zuo Y, Wang C, Zheng X, Liu J (2022). Biochemical and functional characterization of the N-terminal ubiquitin-like domain of human SHARPIN. Protein Expr Purif.

[15] Stieglitz B, Haire LF, Dikic I, Rittinger K (2012). Structural analysis of sharpin, a subunit of a large multi-protein e3 ubiquitin ligase, reveals a novel dimerization function for the pleckstrin homology superfold. J Biol Chem.

[16] Fujita H, Tokunaga A, Shimizu S, Whiting AL, Aguilar-Alonso F, Takagi K, Walinda E (2018). Cooperative domain formation by homologous motifs in hoil-1l and sharpin plays a crucial role in lubac stabilization. Cell Rep.

[17] Walinda E, Morimoto D, Sorada T, Iwai K, Sugase K (2021). Expression, solubility monitoring, and purification of the co-folded LUBAC LTM domain by structure-guided tandem folding in autoinducing cultures. Protein Expr Purif.

[18] Liu X, Luo Y, Li P, Song S, Peng J, Dunbrack RL (2021). Deep geometric representations for modeling effects of mutations on protein-protein binding affinity. PLoS Comput Biol.

[19] Al Quraishi M, Valencia A (2019). Alphafold at casp13. Bioinformatics.

[20] Jumper J, Evans R, Pritzel A, Green T, Figurnov M, Ronneberger O, Tunyasuvunakool K (2021). Applying and improving AlphaFold at CASP14. Proteins.

[21] Ayoub R, Lee Y (2021). Protein structure search to support the development of protein structure prediction methods. Proteins.

[22] Senior AW, Evans R, Jumper J, Kirkpatrick J, Sifre L, Green T, Qin C (2019). Protein structure prediction using multiple deep neural networks in the 13th Critical Assessment of Protein Structure Prediction (CASP13). Proteins.

[23] Callaway E (2020). ‘It will change everything’: DeepMind’s AI makes gigantic leap in solving protein structures. Nature.

[24] Jumper J, Evans R, Pritzel A, Green T, Figurnov M, Ronneberger O, Tunyasuvunakool K (2021). Highly accurate protein structure prediction with AlphaFold. Nature.

[25] Varadi M, Anyango S, Deshpande M, Nair S, Natassia C, Yordanova G, Yuan D (2022). AlphaFold Protein Structure Database: massively expanding the structural coverage of protein-sequence space with high-accuracy models. Nucleic Acids Res.

[26] Jha K, Saha S, Singh H (2022). Prediction of protein–protein interaction using graph neural networks. Sci Rep.

[27] Zhu W, Shenoy A, Kundrotas P, Elofsson A, Cowen L (2023). Evaluation of AlphaFold-Multimer prediction on multi-chain protein complexes. Bioinformatics.

[28] Yu D, Chojnowski G, Rosenthal M, Kosinski J, Cowen L (2023). AlphaPulldown—a python package for protein-protein interaction screens using Alphafold-multimer. Bioinformatics.

[29] Paketurytė V, Zubrienė A, Ladbury JE, Matulis D. Intrinsic Thermodynamics of protein-ligand binding by isothermal titration calorimetry as aid to drug design.
Methods MolBiol 2019, 1964: 61–74. https://doi.org/10.1007/978-1-4939-9179-2_5.

[30] Abraham MJ, Murtola T, Schulz R, Páll S, Smith JC, Hess B, Lindahl E (2015). GROMACS: high performance molecular simulations through multi-level parallelism from laptops to supercomputers. SoftwareX.

[31] Van Der Spoel D, Lindahl E, Hess B, Groenhof G, Mark AE, Berendsen HJC (2005). GROMACS: fast, flexible, and free. J Comput Chem.

